# Beyond Spatial Proximity: Optimizing Nursing Home Bed Allocation with a Proposed ‘4A’ Model—Insights from Guangzhou, China

**DOI:** 10.3390/healthcare14142128

**Published:** 2026-07-15

**Authors:** He Jin, Na Li, Mengya Jia, Mengtian Wu, Shixiong Hu

**Affiliations:** 1 School of Intelligent Transportation and Intelligent Construction Engineering, Huanghe Jiaotong University, Jiaozuo 454950, China; 2025090940@zjtu.edu.cn (H.J.); 2022120101@zjtu.edu.cn (M.W.); 2020052303@zjtu.edu.cn (S.H.); 2 School of Artificial Intelligence, Huanghe Jiaotong University, Jiaozuo 454950, China; 2025010270@zjtu.edu.cn

**Keywords:** elderly care allocation, ‘4A’ model, community-level inequity, optimization–diagnosis–intervention, policy simulation

## Abstract

Background/objectives: As Guangzhou’s population ages rapidly, the supply-and-demand gap in elderly care facilities has become severe. We propose a ‘4A’ model that draws on accessibility, affordability, acceptability, and availability from the classic ‘5A’ access framework to optimize nursing home bed allocation from existing facilities. Methods: We applied the ‘4A’ model to 2630 communities/villages and 243 nursing homes in Guangzhou, using linear programming to maximize a matching score under capacity, demand, and occupancy constraints. Inequality was assessed using the Gini coefficient, Theil index, Moran’s I, and hot spot analysis. Four policy scenarios were simulated under resource-scarce and resource-abundant conditions. Results: A total of 753 (28.6%) communities/villages had no access to nursing homes. The unmet demand rates followed a ‘dual-center’ clustering pattern, and hot spots were not only in the urban core, where ‘scale disadvantage’ and ‘matching disadvantage’ coexisted, but also in outer suburban districts to form a ‘supply vacuum.’ Inequity was moderate, and it originated from between-district disparities. Compared with a ‘distance-only variant’ model, our ‘4A’ model allocated 3813 more beds. Policy simulations showed that minimum service (α=0.1) eliminated all unserved communities/villages; targeted bed expansion increased satisfaction rates to 74.8% and reduced the Gini coefficient to 0.280; and subsidies and quality upgrades became effective only when bed supply was abundant. The four simulations were phased into a three-phase policy roadmap. Conclusions: The ‘4A’ model transforms the qualitative ‘5A’ framework into a computable allocation matrix, offering actionable recommendations for equitable access to elderly care resources.

## 1. Introduction

China is facing the most severe population aging challenge [1,2,3]. In 2023, its elderly population (aged 60 and older) had reached 297 million, accounting for 21% of the total population [4]. As a megacity in China, Guangzhou is facing the same population aging issue; its elderly population was 2.13 million in 2024, a 1.5% increase relative to 2020 [5], and it is predicted to reach 3.2 million by 2030. Despite the rapid growth of the elderly population, the supply of nursing home beds is severely insufficient. In 2025, there were 257 nursing homes with 56,000 beds in Guangzhou, or approximately 26 beds per 1000 elders [6], far below the international standard of 50 beds per 1000 elders. Worse still, the distribution of beds and facilities is extremely uneven [7,8]. The central urban districts have more than 60% of the beds and a dense elderly population; yet, they average only 15–18 beds per 1000 elders. In contrast, the peripheral areas have fewer beds but a sparse population of elders, resulting in a ratio of 20–25 beds per 1000 elders. This structural imbalance (bed shortages in central areas and vacancies in peripheral areas) leaves numerous communities with little or no nursing home bed coverage [7].

Three approaches are commonly used in bed allocation for nursing homes in China, including administrative allocation [9], first-come, first-served [10], and proximity-based allocation [11]. However, these approaches cannot address spatial imbalances or reflect the elders’ true preferences regarding price and service quality. Two-step Floating Catchment Area (2SFCA) and its variants have been widely employed to evaluate the geographic accessibility of healthcare [12,13] and elderly care [14,15]. However, these measures only generate index scores and cannot output specific bed allocation plans. Other studies have used location–allocation models (e.g., p-median) to optimize elderly care location selection [16,17] and bed allocation [18,19], with the objectives of minimizing the total distance or total cost, thereby failing to integrate non-spatial factors (such as price and quality) into their objective functions. Furthermore, Penchansky and Thomas’s ‘5A’ conceptual model provides a critical perspective for understanding access to elderly care [20,21]. Nevertheless, it has not integrated multiple dimensions into a single coherent framework that combines multi-dimensional matching, optimization constraints, and policy simulation.

To bridge these research gaps, we propose a ‘4A’ model that integrates four core dimensions (accessibility, affordability, acceptability, and availability) from the ‘5A’ framework. It determines how many elderly residents from each community should be assigned to each nursing home, subject to bed capacity, demand, and occupancy constraints. We also systematically diagnose the inequity in the allocation outcomes of the ‘4A’ model using various inequity evaluation methods. Lastly, multiple policy scenarios are simulated, and a three-phased plan is provided. This integrated framework, which combines optimization, equity diagnosis, and policy simulation, provides a clear pathway for allocating elderly care beds.

The main goals for this study consist of four parts: (1) constructing a new ‘4A’ model to optimize bed allocation; (2) measuring the degree of spatial inequity of bed service deprivation; (3) simulating multiple policy interventions and comparing how they improve or weaken equity; and (4) proposing an actionable, phased policy plan. Three contributions are identified in this research. Theoretically, we convert the classic ‘5A’ framework into a computable ‘4A’ matching function, transferring our research from a qualitative assessment to quantitative optimization. Meanwhile, we emphasize ‘comprehensive access’ rather than only ‘spatial access’, acknowledging that the dimensions of affordability and acceptability are equal to or no less important than spatial accessibility. Methodologically, we propose a ‘4A’ model and integrate linear programming, spatial statistics, and policy simulation to diagnose inequity and inform intervention strategies. Empirically, our study simulates multiple policy scenarios and provides valuable support for such interventions.

## 2. Literature Review

### 2.1. Spatial Accessibility in Elderly Care Facilities

Spatial accessibility gauges how convenient it is for residents to travel from home to services [22] and serves as a fundamental basis for the spatial allocation of facilities. Early studies often used simple methods, such as shortest-distance or density measures, which failed to capture the complex interactions between the supply and demand sides [22,23]. To address this issue, [12] proposed the 2SFCA, which has been extensively used in academia since then. Subsequent studies introduced distance decay functions (e.g., Gaussian) [13] and other variants [24,25,26,27,28,29] to improve various aspects of the original 2SFCA, and these variants have been widely adopted.

In the field of elderly care, the 2SFCA and its variants have been widely used to measure spatial accessibility for several cities. For example, Ref. [14] performed a community-level analysis in Xi’an and found higher accessibility in the south-central areas. Ref. [8] conducted community- and street-level analyses in Guangzhou and showed that central areas such as Yuexiu and Tianhe had higher accessibility than outer suburbs at both scales. These studies reveal a consistent pattern—the spatial accessibility is higher in urban and central areas but lower in suburban and peripheral areas. However, these studies have two limitations. First, they focused solely on ‘spatial distance’ but largely ignored elders’ preferences for affordability and service quality in nursing homes; seniors weigh not only spatial proximity but also a reasonable price and good quality. Second, these studies only output accessibility indices that are useful for comparing high- and low-access areas but fail to answer the question ‘How many beds should be allocated to which communities from which nursing homes?’ Our ‘4A’ model addresses both limitations by incorporating spatial accessibility, affordability, and acceptability into the objective function, yielding a concrete bed allocation matrix.

### 2.2. Location-Allocation Optimization for Elderly Care Facilities

Optimization methods for elderly care facilities can be broadly classified into three categories: efficiency-oriented, equity-oriented, and multi-objective models [30].

Efficiency-oriented models aim at minimizing the total travel distance or cost [31]. The p-median problem is the most prominent model and has been utilized in many studies across different countries. Applications include a hierarchical and modified p-median problem in Japan [32,33], a branch-and-bound algorithm in South Korea [34], a hierarchical multi-service mathematical programming model in Portugal [35], and mixed-integer linear programming in the United States [36]. While these methods prioritize maximizing system efficiency, they often neglect equity in rural and low-demand areas.

Equity-oriented models aim to optimize equitable access in remote areas for disadvantaged groups. Common approaches include introducing the Rawlsian criteria [37] (e.g., mandating a minimum service for even the worst-served community to receive certain access), proposing optimization algorithms (i.e., a particle swarm optimization algorithm [38]), and imposing equity constraints [39,40] (e.g., Gini coefficient and Lorenz curves). These models improve the overall equity of access but are achieved at the cost of model efficiency, rarely taking non-spatial factors such as affordability and quality into consideration.

Multi-objective models represent trade-offs between efficiency and equity. Studies in Shanghai, China, optimized two objectives by either considering the total distance and construction costs [41], or economic and welfare constraints [42]. Others balanced three objectives (e.g., efficiency, travel costs, and profits) using a modified immune algorithm [43]. Complex and advanced algorithms are incorporated into more sets of objectives [44]. These models are more flexible than efficiency-oriented and equity-oriented models. However, they mostly focus on locating new facilities rather than allocating beds from existing ones, failing to incorporate operational constraints such as minimum and maximum occupancy rates.

Several studies have addressed healthcare capacity allocation using multi-objective approaches [45,46]. Unlike these models, our ‘4A’ model is a single-objective optimization model that integrates multiple dimensions into a single composite objective function. A closely related work by [47] developed a mathematical framework for regional hospital case mix planning that optimally assigns patients to hospitals based on distance and travel time with bed capacity constraints, providing a methodological reference for our allocation model, although our approach focuses specifically on nursing home bed allocation.

Despite these advances, three gaps remain. First, these methods are primarily for selecting optimal locations (e.g., determining where to build new facilities) instead of allocating existing beds. Second, current methods rarely incorporate operational constraints such as minimum and maximum occupancy rates, resulting in optimization outcomes that are difficult to implement in practice. Third, optimization objectives primarily focus on spatial aspects (e.g., travel distance and coverage) but neglect non-spatial aspects such as affordability and acceptability. Our ‘4A’ model fills these gaps by allocating beds from existing facilities under capacity, demand, and occupancy constraints, and integrating affordability and quality into the objective function.

### 2.3. Affordability and Acceptability as Barriers to Accessing Elderly Care

The ‘5A’ framework emphasizes affordability and acceptability as equally important as spatial accessibility. Affordability reflects economic barriers: when a nursing home’s fee exceeds an older person’s ability to pay, even though the physical distance is short and beds are sufficient, they still lack access to these facilities. Acceptability measures the match between service and personal preferences; if a nursing home’s service quality or medical services do not meet elders’ expectations, they may refuse admission.

Empirical studies show that affordability influences facility choices; high-income elders in Zhejiang province, China, were more likely to prefer higher-level services [48]. Affluent Japanese seniors favored facilities offering higher-cost services, whereas low-income older adults preferred nursing homes for the lowest budgets [49]. Price itself also matters when choosing facilities; the probability of choosing a nursing home decreased significantly as both distance and price increased [50]. In terms of quality, its influence is inconsistent. Ref. [50] in Germany showed that service quality was not statistically significantly associated with nursing home choices. But a study from the United States [51] found that one-star nursing homes lost 8% of their market share, while five-star nursing homes gained over 6% after the introduction of a Five-Star Quality Rating System. Although these studies acknowledged price and service quality as barriers, they rarely integrate these dimensions into resource allocation models.

### 2.4. Policy Simulations in Elderly Care Resource Allocation

Policy simulation is a powerful tool to assess the impacts of different resource allocation scenarios. Few elderly care studies have evaluated the impact of simulation scenarios at the macro level, including a stochastic multi-objective model [52], a multi-agent system simulation [53], a system dynamics model [54], and a ‘forecasting–simulation–optimization’ framework [55].

These policy simulations, however, are mostly conducted at the aggregate level. There is a lack of micro-level simulations, such as capacity expansion around completely unserved communities, price subsidies for low-income neighborhoods, or quality upgrades for poorly rated facilities. Moreover, their simulation results fail to phase into short-, medium-, and long-term plans; their policy only tells stakeholders ‘what’ to do but does not provide a sequence of ‘what to do first, then what next’.

### 2.5. ‘4A’ Model: A Decision-Oriented Allocation Framework

To emphasize the novelty and positioning of the proposed ‘4A’ framework, we differentiate it from three common types of existing methods. Table 1 presents a comparison, revealing that the ‘4A’ model has several advantages over existing ones. First, it outputs a concrete bed allocation matrix $X_{ij}$, which represents the number of beds allocated from community i to nursing home j, rather than spatial accessibility indices as 2SFCA and its variants, or facility locations as location-allocation models. Second, it directly integrates affordability and acceptability into the objective function, whereas the other three methods typically do not. Third, it enforces realistic constraints, such as capacity, demand (3% of the elderly population), and occupancy constraints (10–95%); other methods impose no or partial constraints. These characteristics make the ‘4A’ model stand out as a unique, decision-oriented allocation framework for guiding actionable plans for equitable access to elderly care services.

## 3. Materials and Methods

### 3.1. Study Area and Data Source

**Figure 1:**
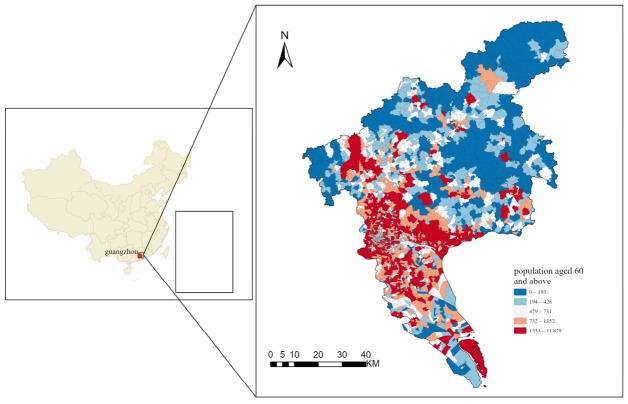
Study area: a graduated-color map of the distribution of the elderly population across 2630 communities/villages in Guangzhou.

Guangzhou, capital of Guangdong Province, administers 11 districts, 177 towns/sub-districts, and over 2600 communities/villages. By 2024, its elderly population had reached over 2 million (18.5% of the total). The unit of analysis is at the community/village level. Communities/villages are the smallest census units in China with clear geographical boundaries and demographic data. Figure 1 shows that most elders are distributed in the central urban districts, such as Haizhu, Yuexiu, Liwan, and Tianhe.

Elderly population data for 2630 communities/villages were retrieved from the Seventh National Population Census and Statistical Yearbooks. Community/village-level monthly income data were obtained from the Guangzhou Statistical Yearbook based on the National Household Survey. We excluded 12 communities/villages with zero elderly population and missing income data. We did not use the total elderly population directly, as this would overestimate effective demand. Instead, we adopted a ‘9073’ elderly care framework used by the Chinese government: 90% of seniors are cared for at home, 7% in communities, and 3% in institutions. Thus, effective demand in each village/community was calculated as 3% of the total elderly population. Note that we also tested alternative demand ratios (2%, 2.5%, 3%, 5%, and 10%); the results are provided in Appendix A, Table A3.

We obtained nursing home data from the Guangzhou Civil Affairs Bureau, which releases annual data on senior care facilities. We collected data on 243 nursing homes, including their names, addresses, beds, types, and whether they provide medical services (e.g., rehabilitation, on-site clinics, and hospitals). The nursing home’s monthly price was purchased through a third-party data company. We found that 8 of 243 facilities (3.3%) had missing price data, which were imputed using the median of facilities with the same ownership type and district. Given the small proportion of missing data, the imputation is unlikely to introduce substantial bias. Star ratings (1-star through 5-star) for nursing homes are also available, but are published on different pages. Only 52 nursing homes have star-rating data, and we used them to represent service quality. For those without star ratings, we created service quality scores by ownership types (e.g., private, private high-end, and private ordinary). A sensitivity analysis was conducted by adjusting the imputed quality scores by ±20% to assess uncertainty (see Appendix A, Table A4).

**Figure 2:**
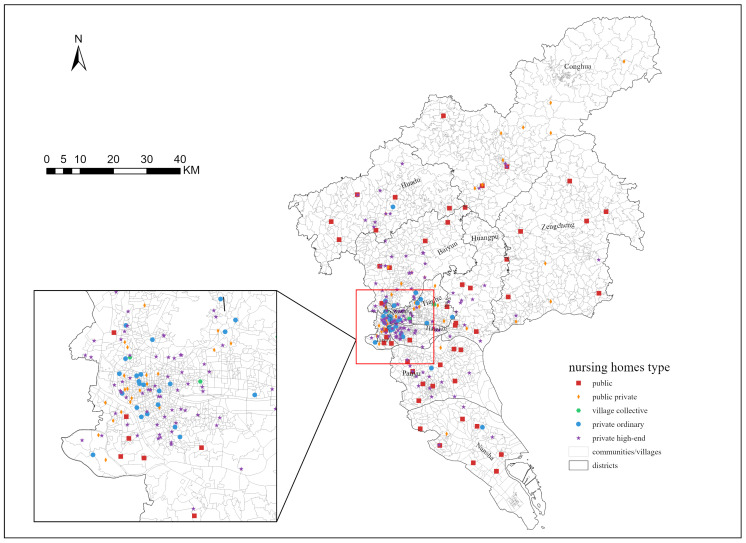
Spatial distribution of 243 nursing homes and their types; ‘public private’ in the legend refers to facilities that are publicly owned but privately operated.

We used the Gaode web service API (version 3) to geocode nursing home addresses; 241 out of 243 addresses were initially matched. After correcting format errors for the two unmatched records, all 243 nursing homes were successfully geocoded. To verify the positional accuracy of geocoding results, we manually checked 25 matched points (about 10% of the total) against satellite imagery in ArcGIS Pro 3.5.4. We found that the median positional error was approximately 20 m, which is negligible considering the distances used in this study (5–30 km). Figure 2 shows that most nursing homes are located in central urban districts, but communities/villages in outer suburban districts are severely lacking in nursing homes.

Guangzhou road Shapefile was collected from OpenStreetMap. Then, we created a network dataset in ArcGIS Pro 3.3 and calculated the OD distance from each village/community (using the population-weighted center) to a nursing home, with travel distance in meters as cost. This generated a 640,000 OD distance matrix (2630 × 243) and was used in subsequent calculations. In total, 15 remote communities/villages had no network path within 30 km and were treated as having zero spatial accessibility.

Potential measurement errors may come from sampling errors in community/villages income data from the National Household Survey. However, such errors are likely consistent across communities. Monthly income was obtained via a third-party provider and may contain inaccuracies; we acknowledge this as a limitation in Section 6. Geocoding errors are minimal (20 m).

### 3.2. Theoretical Framework of This Research

**Figure 3:**
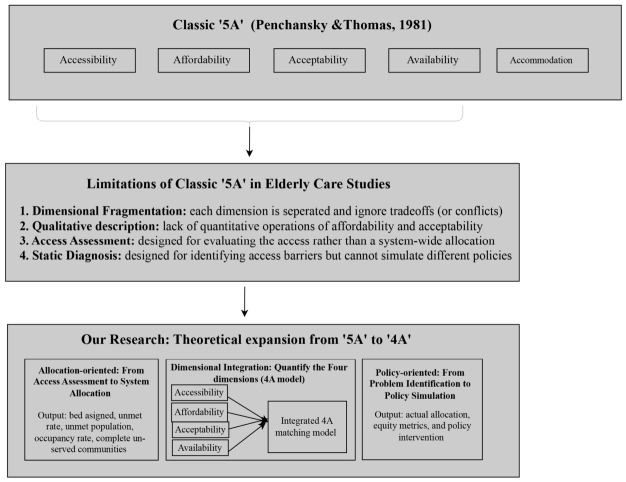
Theoretical framework [56].

Figure 3 presents the theoretical framework for our study. Penchansky and Thomas [56] defined healthcare access through five dimensions: Accessibility (geographic proximity), Availability (adequate supply of services), Affordability (price relative to an individual’s ability to pay), Acceptability (match between patient preferences and provider characteristics, e.g., language, culture, and quality), and Accommodation (organizational features such as hours and appointment systems). The ‘5A’ framework serves as a basis, and we use this classic theory to examine its application in nursing home bed allocation. We adopt four of these dimensions but exclude accommodation (e.g., operating hours, room types, and disability support) due to data unavailability. This exclusion may create bias for populations with special needs, but Guangzhou’s elder care system is reasonably standardized. For instance, the national five-star rating system defines uniform safety and occupancy standards for all licensed nursing homes. This baseline ensures that some basic accommodation needs of older people are met. Although bias persists, its effect is limited. Furthermore, our quality score (Equation (3), serving as a proxy for acceptability) includes medical services, which correlates with some accommodation elements. We will obtain accommodation data and integrate it into our model in the future. We identified four limitations in the ‘5A’: (1) its five dimensions are processed separately, ignoring the trade-offs among them; (2) three dimensions remain on qualitative descriptions (except for the accessibility and availability), lacking quantitative operations on them; (3) it is designed for an access evaluation rather than a system allocation under multiple constraints, and (4) it is usually used for the problem identification (e.g., what and where the barriers are) but cannot simulate the effects of varying resource conditions. To overcome these constraints, we propose a ‘4A’ model that integrates the four core dimensions into a matching score function. The ‘4A’ model expands the classic ‘5A’ in three aspects: dimensional integration, allocation orientation, and policy orientation (see Figure 3 for details).

### 3.3. Analytical Workflow of This Study

The analytical workflow contains three steps: (1) ‘4A’ model development, (2) inequity analysis, and (3) policy simulation. These steps form an integrated optimization–diagnosis–intervention model. We first quantify each dimension and integrate them into a matching score function to compute the matching score of each nursing home. We then conduct allocation optimization (see Section 3.4 for details) to maximize the total matching score subject to bed capacity constraints and lower and upper bounds on occupancy rates. The optimization results are reported. The second step conducts a series of inequity checks: the Gini coefficient, Theil index, Global Moran’s I, and Getis–Ord Gi* analysis. Finally, we perform four policy simulations (Section 3.6) and finalize a three-phased intervention plan aimed at eliminating unserved communities and reducing inequity over short-, medium-, and long-term time frames.

### 3.4. A ‘4A’ Optimization Model

Our optimization framework adapts the logic of regional capacity allocation models [47] to nursing homes by integrating the ‘4A’ dimensions (accessibility, availability, affordability, and acceptability) into the objective function and optimizing bed allocations under multiple constraints.

**Figure A1:**
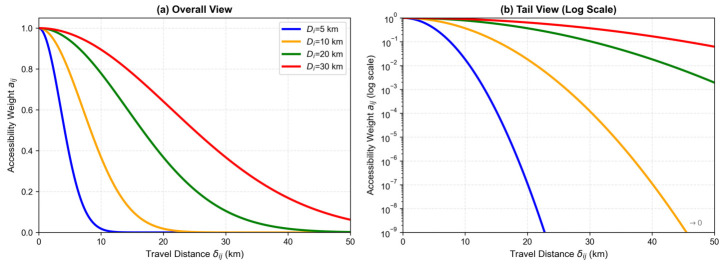
Gaussian distance decay function for different $D_{i}$ values: (**a**) overall view using linear scale, showing the monotonic decreasing relationship between $a_{ij}$ and travel distance $\delta_{ij}$; and (**b**) tail view details using log scale, revealing that $a_{ij}$ asymptotically approaches 0 but remains positive ($a_{ij}>0$) for all finite distances. The notation “→ 0” in (**b**) indicates that the value of $a_{ij}$ tends toward zero but never actually reaches it.

The first step is to turn the four core dimensions into a measurable ‘4A’ matching function. Below are specific quantifications for each dimension:

(1) Accessibility. We use the Gaussian distance decay function (smooth decay, commonly used in 2SFCA variants [13]) to measure spatial accessibility with the network distance as the travel resistance. See Equation (1):

(1) $$a_{ij}=\;\exp\left(-{\left(\frac{\delta_{\mathrm{ij}}}{D_{i}}\right)}^{2}\right)\;if\;\delta_{ij}<\;\infty ;\;\mathrm{otherwise},a_{ij}\;=0$$

where $\delta_{\mathrm{ij}}$ is the network distance between village/community i and nursing home j; $D_{i}$ denotes the maximum travel distance threshold for the district in which community/village i is located. $a_{ij}$ decreases monotonically as $\delta_{ij}$ increases: $a_{ij}=1$ when $\delta_{ij}=0$, and $a_{ij}\to 0$ as $\delta_{ij}\to \infty$. Appendix B, Figure A1 visualizes this distance decay function for different $D_{i}$ values, showing the overall decay trend (a) and the asymptotic tail behavior (b). We set different $D_{i}$ values: 5 km for central districts (Yuexiu, Tianhe, Haizhu, and Liwan), 10 km for suburban districts (Baiyun, Huangpu, and Panyu), 20 km for outer suburban districts (Huadu and Nansha), and 30 km for rural districts (Conghua and Zengcheng). The distance thresholds (5 km, 10 km, 20 km, and 30 km) were derived based on the time-based service circle standards [57].

(2) Affordability is represented as a ratio $b_{ij}$ (Equation (2)):

(2) $$b_{\mathrm{ij}}=\frac{{\mathrm{income}}_{i}}{{income}_{i}+{price}_{j}}$$

where ${\mathrm{income}}_{i}$ denotes the average monthly income of community/village i; ${price}_{j}$ is the monthly price for nursing home j; and its value range is (0, 1]. A larger value indicates greater affordability of nursing home j for community i. Note that an alternative affordability measure (1 − price/income) is more intuitive when price < income, but it becomes negative when price > income. Our formulation avoids this issue and ensures affordability values in (0, 1]; a combination of the two formulae will be explored in future work.

(3) Acceptability. We define service quality $q_{j}$ as a proxy of acceptability (Equation (3)):

(3) $$q_{j}={\alpha \;}_{1}\;\times \;{\mathrm{star}\text{\_}\mathrm{part}}_{j}+{\alpha \;}_{2}\;\times \;{\mathrm{size}\text{\_}\mathrm{score}}_{j}+{\alpha \;}_{3}\;\times \;{\mathrm{medical}\text{\_}\mathrm{score}}_{j}$$

where ${\alpha \;}_{1},$ ${\alpha \;}_{2},$ and ${\alpha \;}_{3}$ are weights of each component. We set the three weights at ${\alpha \;}_{1}=0.7$, ${\alpha \;}_{2}=0.15$, and ${\alpha \;}_{3}=0.15$ to reflect the relative importance of each component; star rating is assigned a larger weight (0.7) because it is systematically evaluated by government authorities and is the most reliable quality indicator in China. Each component (${\mathrm{star}\text{\_}\mathrm{part}}_{j}$, size_score, medical_score_j_) is scaled to [0, 1] by construction (see definitions and equations below):

- ${\mathrm{star}\text{\_}\mathrm{part}}_{j}$ is the star rating for nursing home j. However, only 52 out of 243 nursing homes in Guangzhou had a star rating by 2025. The ${\mathrm{star}\text{\_}\mathrm{part}}_{j}$ contains two parts (see Equation (4)). For those with star ratings, star_score_j_ is directly assigned: five-star = 1.0, four-star = 0.9, three-star = 0.7, two-star = 0.5, one-star = 0.4, and no-star = 0.2; For those without star ratings, we used ownership types as a proxy: public–private = 0.9, private high-end = 0.8, public = 0.7, private ordinary = 0.4, and village-collective = 0.3:
  (4) $${star\_part}_{j}=\left\{\begin{array}{ll} {star\_score}_{j}, & ifstarratingexists\\ {owner\_score}_{j}, & otherwise \end{array}\right.$$
- ${size\_score}_{j}$ is the capacity of nursing home j; a logarithmic transformation turned its value into (0, 1] (Equation (5)). ${Max}_{beds}$ is the maximum bed count among all nursing homes:
  (5) $${size\_score}_{j}=Log({beds}_{j}+1)/Log({Max}_{beds}+1)$$
- medical_score_j_ represents whether a nursing home j provides medical service. We assign values: facilities with rehabilitation and/or hospitals = 1.0, on-site clinics or designated as medical insurance facilities = 0.7, and others = 0.2.

(4) Availability is represented as effective capacity C_j_ effective (Equation (6)):

(6) $$C_{j}\mathrm{effective}=\;(0.40+0.55q_{j})\;\times \;C_{j}$$

where $C_{j}$ is the physical bed capacity and $q_{j}$ is the quality score for nursing home *j*. The range (0.40–0.95) is based on practical reasoning that higher-quality facilities would sustain higher occupancy rates. The 40% lower bound reflects a typical occupancy threshold; below this level, a nursing home would struggle to operate. The 95% upper bound accounts for bed turnover and other factors.

We assemble the ‘4A’ matching function $M_{\mathrm{ij}}$ as a weighted sum of three dimensions: spatial accessibility $a_{ij}$, affordability $b_{ij}$, and service quality $q_{j}$ (Equation (7)):

(7) $$M_{ij}=w_{1}\;\times \;a_{ij}+w_{2}\;\times b_{ij}+w_{3}\;\times \;q_{j}$$

where $w_{1}$, $w_{2}$, and $w_{3}$ are positive weight coefficients. We assigned baseline weights as follows: $w_{1}=0.2$, $w_{2}=0.$4, and $w_{3}=0.$4. The weights were chosen based on the ‘4A’ framework, which views affordability and acceptability as no less important than spatial accessibility. A lower weight (0.2) was assigned to spatial accessibility because our model allocates beds from existing nursing homes with fixed locations; there is no need to select new sites. Under this context, although spatial distance still matters, empirical evidence suggests that, once facilities are within a reasonable travel range, price and quality become more critical than distance differences [48,50]. We tested a range of alternative weight combinations (see Appendix A, Table A1), and the baseline weights (0.2, 0.4, and 0.4) achieved the best balance between satisfaction and equity. To avoid unreasonable assignments, we use a matching threshold θ=0.3 in the baseline model, meaning that O-D pairs with $M_{ij}\geq 0.3$ are allocated.

The second step is to build a capacity-constrained optimization model. We formulate an objective function Z to maximize the matching score over all community/village–nursing home pairs (Equation (8)).

(8) $$MaxZ=\sum_{i}\sum_{j}M_{ij}\;\times X_{ij}$$

where $X_{ij}$ is a decision variable and represents the number of elderly people allocated from community/village i to nursing home j; and $X_{ij}$ collectively forms a matrix of size I (communities/villages) by J (nursing homes), which is the direct output of the optimization model.

The optimization is subject to the following constraints:

Capacity constraint:

(9) $$\sum_{i}X_{\mathrm{ij}}\leq C_{j}effective$$

Demand constraint:

(10) $$\sum_{j}X_{\mathrm{ij}}\leq d_{i}$$

Occupancy constraints:

(11) $${0.10\leq o}_{j}\leq 0.95$$

Non-negativity:

(12) $$X_{\mathrm{ij}}\geq 0$$

where $C_{j}\mathrm{effective}\;=\;(0.40+0.55q_{j})\;\times \;C_{j}$;$\;d_{i}$ is the effective demand of community i; and occupancy constraints mean that each nursing home must operate within a feasible occupancy range (0.10–0.95).

The ‘4A’ model was implemented in Python 3.9 in Anaconda using the PuLP package (version 2.7.0) for modeling. We used an open-source HiGHS solver (version 1.5.1) for a linear programming optimization with the default settings. The optimization created 2630 × 243 = 639,090 OD pairs; due to the matching threshold (θ > 0.3), 439,530 decision variables ($X_{ij}$) were retained in the analysis. The solution time was approximately 3 min using an Intel Core i7-7240H processor (Intel Corporation, Santa Clara, CA, USA) with 32 GB RAM. For larger metropolitan regions, the computation would scale approximately linearly with the number of communities/villages and nursing homes, so our model should remain efficient for most regional-level applications. For national-scale data, the computational complexity will increase substantially; decomposition or heuristic methods should be used. The Python code is available upon request for reproducibility.

The final output includes: allocated beds (${\mathrm{ab}}_{i}$) and unmet demand rate ($u_{i}$) for each community/village i, as well as occupancy rate ($o_{j}$) for each nursing home j. See their respective Equations (13)–(15) below. Moreover, an overall satisfaction rate SR was computed for all villages/communities (Equation (16)):

(13) $${\mathrm{ab}}_{i}\;=\;\sum_{j}X_{\mathrm{ij}}$$

(14) $$u_{i}=\frac{d_{i}-{\mathrm{ab}}_{i}}{d_{i}}\times 100$$

(15) $$o_{j}=\frac{\sum_{i}X_{\mathrm{ij}}}{C_{j}}\times 100$$

(16) $$SR=\frac{\sum_{i}\sum_{j}X_{ij}}{\sum_{i}d_{i}}\times 100$$

### 3.5. Inequity Analyses

To assess spatial clustering and identify hot spots of unmet demand, we applied Moran’s I and Getis–Ord Gi. To measure whether the overall allocation is equitable, we calculated the Gini coefficient $G_{i}$ [58]. However, the Gini coefficient only shows overall inequity and does not reveal its sources [58]. We therefore computed the Theil index, as it can decompose inequity into between- and within-group components. Their formulae are provided in Appendix A, Equations (A1)–(A7).

### 3.6. Policy Simulation Design

Based on the baseline allocation results and the identified CUC mechanisms (supply vacuum, scale disadvantage, and matching disadvantage), we designed four policy scenarios to address these deprivations: (1) minimum service level, (2) targeted bed expansion, (3) price subsidy, and (4) quality upgrade. The configuration of each scenario is described below:

Scenario I: Minimum service level (α = 0.1). We implemented this scenario to ensure that each community/village would receive at least 10% of its effective demand in beds, as recommended by [37].

Scenario II: Targeted bed expansion. We increased bed capacity by 20% in nursing homes within 5 km of completely unserved communities/villages (CUCs), assuming that the bed expansion occurs within existing facilities (e.g., adding floors, converting unused space, or installing add-on units) rather than through new construction.

Scenario III: Price subsidy. We offered a 20% discount on nursing home prices for elderly residents in low-income communities/villages, defined as having a monthly income below the sample median (5000 CNY, approximately 700 USD).

Scenario IV: Quality upgrade. We increased the quality of facilities with scores below 0.3 by 0.2.

For each policy scenario, we modified the corresponding input parameters as described above, then re-ran the linear programming optimization in Python using the same PuLP model and HiGHS solver as in the baseline. Section 4.4 reports each scenario’s satisfaction rates, Gini coefficients, CUC counts, and their changes relative to the baseline.

## 4. Results

### 4.1. Baseline Allocation Results

Under the baseline matching threshold (θ = 0.3) and four constraints—bed capacity constraint ($\sum_{i}X_{\mathrm{ij}}\leq C_{j}effective$), demand constraint ($\sum_{j}X_{\mathrm{ij}}\leq d_{i}$), occupancy constraint (${0.01\leq O}_{j}\leq 0.95$), and no negative constraint ($X_{\mathrm{ij}}>0$)—the linear programming optimization maximizes the total matching score (Equation (8)). It outputs a total allocation of 48,553 beds for 70,686 older adults, yielding a satisfaction rate of 68.7% (total allocated beds/total effective demand—Equation (13)) (Table 2). The Gini coefficient is 0.33, a moderate level of overall inequality. We found that 15 communities/villages have no OD connection, as no nursing homes are within the maximum travel distance of 30 km; and 753 Communities/villages are not assigned any beds, accounting for 28.60% of the total. To further characterize these 753 CUCs, we examined certain demographic characteristics in these areas. The median income is 6381 (Q1: 5043, Q3: 7757) CNY, the median elderly population is 18.0 (Q1: 7.2, Q3: 37.2) persons, and the median elderly density is 35 (Q1: 2, Q3: 216) persons/km^2^. Among CUCs, 41.5% are rural, and 58.5% are urban.

**Figure 4:**
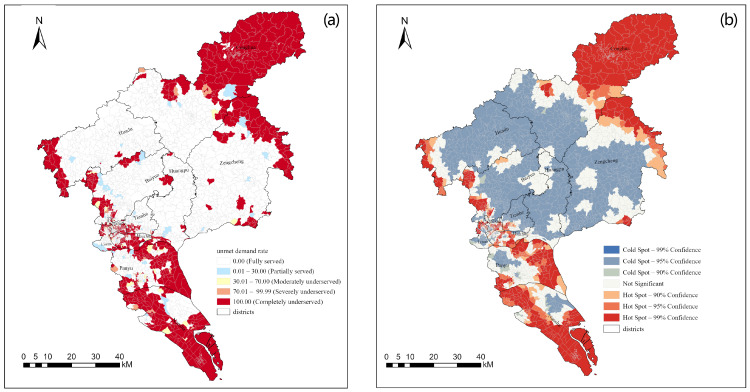
(**a**) Spatial distribution of unmet rate; and (**b**) hot spot analysis of unmet rate.

The unmet demand rate, u_i,_ is the percentage of effective demand that is not satisfied (see Equation (14)). Figure 4a shows the distribution of unmet demand rates for all communities/villages. About 65% of communities/villages are fully served (0%, white) and concentrated in Huangpu, Panyu, Huadu, and most of Zengcheng. Overall, the unmet demand rates do not follow the conventional ‘high in the center, low in the periphery’ pattern. Instead, they exhibit a ‘dual-center’ clustering pattern. High unmet rates (dark red, unmet = 100%) are located in two areas: (1) the peripheral fringes, including the northeastern Conghua, the margins of Zengcheng, and the outer part of Nansha; and (2) the core of central urban districts, most of Haizhu and Yuexiu, southern Tianhe, and eastern Liwan. This pattern reveals that elderly care service deprivation exists in both central and peripheral areas of Guangzhou through different mechanisms, which we will discuss in depth in Section 5.

The value of global Moran’s I is 0.567 (Z = 61.36, *p* < 0.01), illustrating a statistically significant clustering pattern of unmet demand rates in the study area. Figure 4b presents the hot spot analysis, verifying the ‘dual-center’ clustering pattern observed in Figure 4a with 99% confidence.

**Figure 5:**
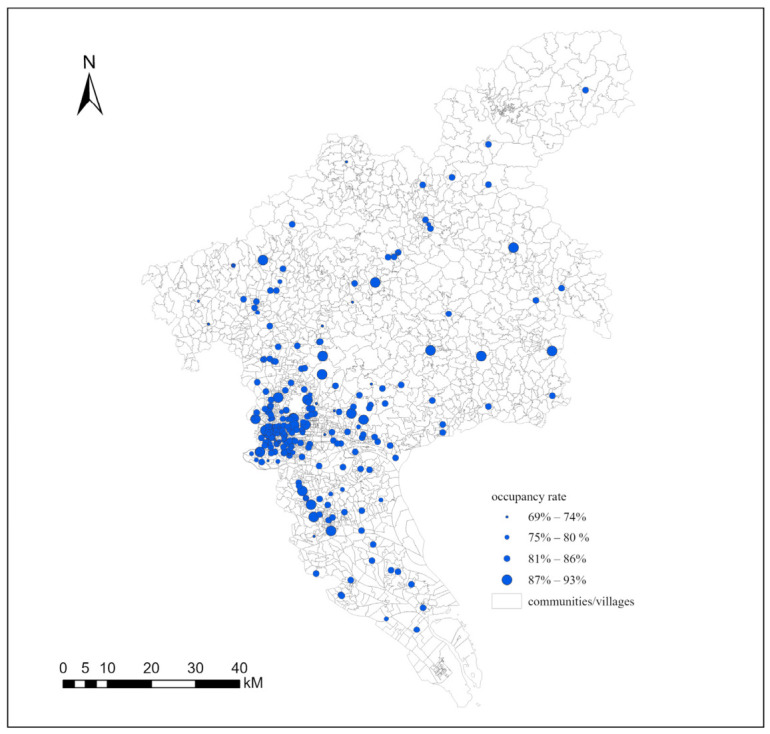
Occupancy rates of nursing homes in Guangzhou.

Nursing home occupancy rates are shown in Figure 5. The mean occupancy rate is 84.3% (SD = 4.5%); high occupancy rates (87–93%) are observed in the central urban districts (Yuexiu and Haizhu) and suburban districts of Baiyun and Panyu. Low occupancy rates (69–74%) are scattered, mostly on the fringes of outer suburbs. Overall, occupancy rates in central urban and near-suburban districts are higher than those in outer suburbs. We compared Figure 5 with Figure 4a and found that central urban areas have numerous CUCs but high occupancy rates, suggesting a mismatch between resource distribution and service needs.

### 4.2. Inequity Results

The inequity analysis indicates a moderate level of inequity (Theil index 0.350), with 92.9% of the inequity attributed to within-district disparities and only 7.1% to between-district disparities. This decomposition indicates that the overall inequity is mainly driven by polarization within the same districts; some communities/villages are well-served while others are completely unserved.

### 4.3. Impact of Adding Affordability and Quality

To highlight the effects of affordability and quality, we compared the ‘4A’ model ($w_{1}$ = 0.2, $w_{2}$ = 0.4, and $w_{3}$ = 0.4) against a ‘distance-only variant’ model ($w_{1}$ = 1, $w_{2}$ = 0, and $w_{3}$ = 0) under three matching thresholds (0.0, 0.3, and 0.5). Note that the ‘distance-only variant’ is essentially a sensitivity test within the same optimization structure; only the weights differ. Table 3 reports the comparison result. At the baseline threshold of 0.3, the ‘4A’ model allocates 48,553 beds, whereas the ‘distance-only variant’ allocates only 47,137 beds. Our ‘4A’ model with affordability and quality improves equity slightly (Gini: 0.330 vs. 0.387; SR: 68.7% vs. 66.7%). At a higher threshold of 0.5, the ‘4A’ model retains the same allocation (48,553 beds), but the ‘distance-only variant’ drops sharply to 44,740 beds.

### 4.4. Policy Simulation Results

All four policy simulations were conducted with a matching threshold of 0.3. Table 4 presents the results. The minimum service scenario does not change the satisfaction rate (68.70%) or the number of allocated beds (48,553) compared with the baseline model; however, it eliminates all CUCs (from 753 to 0) and improves the Gini coefficient from 0.330 to 0.316. The target bed expansion increases the number of allocated beds from 48,553 to 52,891, raising the satisfaction rate to 74.8%. The Gini coefficient drops to 0.280. The number of CUCs decreases by 121. Under the price subsidy scenario, the total allocated beds and satisfaction rate remain unchanged, but the Gini coefficient improves by 0.013, and CUCs decrease by 32. The price subsidy policy appears less effective under the resource-tight condition. Similarly, the price subsidy only slightly increases the number of allocated beds and improves the satisfaction rate by 0.5%, while the Gini coefficient only drops by 0.008. Like the price subsidy, quality upgrades have limited effects under the current conditions.

**Figure A2:**
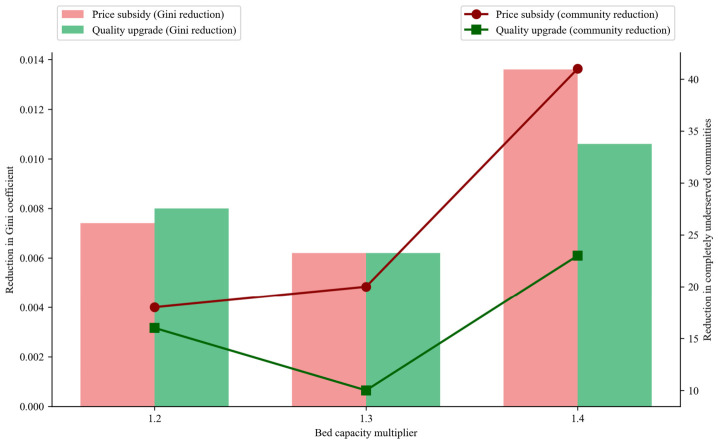
Marginal effects of price subsidy and quality upgrade under resource-abundant conditions.

Given that the effects of price subsidies and quality upgrades are limited under current resource-scarce conditions, we examined their performance under resource abundance (e.g., bed capacity increases by 120%, 130%, and 140%); see results in Appendix A, Table A2. As the bed capacity increases, the satisfaction rate rises from 82.34% to 96.08%, while the Gini coefficient drops from 0.213 to 0.121, and CUCs drop from 441 to 195. Both price subsidies and quality upgrades provide additional equity benefits, and the effects become stronger as resources become abundant (Appendix B, Figure A2). In addition, the sensitivity analysis in Appendix A, Table A3, shows that the satisfaction rates and CUCs vary with demand ratios (2%, 2.5%, 3%, 5%, and 10%), but the ‘dual-center’ pattern and three CUC mechanisms remain robust.

## 5. Discussion

### 5.1. The ‘Dual-Center’ Pattern of Unmet Demand Rates and Their Mechanisms

Our analysis reveals a ‘dual-center’ pattern of elderly care deprivation in Guangzhou. Ref. [8] showed a ‘high in the center, low in the periphery’ pattern using the 2SFCA method. Our findings do not contradict [8] as their study identified where nursing homes were located and whether they were spatially accessible, while ours explains why elderly care demands are not met in urban centers and peripheral communities/villages due to the formation of a ‘scale disadvantage’, ‘matching disadvantage’, and ‘supply vacuum’.

The ‘dual-center’ clustering of unmet demand rates reveals two different mechanisms. We analyze these mechanisms separately for central urban and peripheral suburban districts, as explained below:

1. The ‘supply vacuum’ in peripheral suburban districts

Nursing homes in peripheral suburban districts, such as Conghua, Zengcheng, and Nansha, have a scarcity of facilities. The average distance from a community/village to the nearest facility is about 15 km. When the maximum travel distance $D_{i}$ is set to 30 km, the spatial accessibility becomes quite small (<0.01), resulting in its limited contribution to the total matching scores. Due to a shortage of nearby facilities, these communities/villages cannot receive any bed allocation, even though there is demand in these areas. We thus categorize it as a ‘supply-vacuum’ pattern. See Table 5 for a summary.

2. The ‘scale disadvantage’ + ‘matching disadvantage’ in central urban districts

Central urban districts have dense concentrations of elderly care facilities and relatively large numbers of beds; yet, we still found numerous CUCs there. These CUCs could be categorized into low- and high-effective-demand types.

CUCs with a low effective demand (1–5 people): These communities/villages have quite a small demand, as only one to five older people seek facility care. Our ‘4A’ model maximizes the total matching score and prioritizes bed allocation to the higher-demand elderly when the bed capacity is limited. Moreover, CUCs with a low effective demand are often located on the fringes of central urban areas, where nearby facilities may have already been occupied by high-demand communities/villages. We thus characterize a ‘scale disadvantage’ pattern because the elderly population is so small and cannot compete against larger communities/villages (Table 5).

CUCs with a high effective demand (>15 people): These communities/villages have a greater effective demand but lower matching scores. Recall that, in our model, spatial weight accounts for only 0.2; affordability and quality together account for 0.8. Although a community/village has a high effective demand, if its residents’ incomes are low or the quality of nearby nursing homes is low, its total matching score may not be high. The lower matching scores are caused by two factors: (1) Limited ability to pay: The monthly income in CUCs is lower than the fees at nearby facilities. Central urban nursing homes generally have higher fees, further worsening the elders’ ability to pay. (2) Poor facility quality: The quality scores of nearby institutions are so low that their contribution to the overall matching score is minimal. We categorize it as a ‘matching disadvantage’ pattern, which describes communities/villages having a high demand but losing attractiveness in terms of price and quality.

The demographic characteristics of CUCs reported in Section 4.1 further support the three mechanisms. The urban–rural composition (58.5% urban vs. 41.5% rural) reflects the ‘dual-center’ pattern: some CUCs are in central urban areas; others are in peripheral rural areas. The ‘scale disadvantage’ and ‘matching disadvantage’ are more relevant in urban settings (median elderly population of 18 persons; small elderly population) and a moderate income of 6381 CNY. The ‘supply vacuum’ is more associated with rural settings, where the low elderly density (35 elders/km^2^) does not favor facility siting.

### 5.2. Policy Implication and a Three-Phased Plan

Table 5 provides direct support for a refined phased intervention plan, illustrated below:

Phase 1—Short-term (0–5 years): A minimum service constraint (α = 0.1) eliminates all CUCs. It is achieved by adding new beds through reallocating existing beds from nearby over-served communities/villages to unserved ones. It reduces the satisfaction rate of previously well-served communities/villages but ensures a minimum level of service for all. Implementation methods include the following: (a) providing subsidies to institutions that meet the quota; and (b) mandating the quota for each community/village, and failing to comply with the quota results in penalties.

Phase 2—Medium-term (6–10 years): Targeted bed expansion can address the ‘supply-vacuum’ and ‘matching-disadvantage’ CUCs. A good implementation measure is to increase the number of beds by 20% within 5 km of CUCs, which would require approximately 2500 additional beds. This generally needs a few urban renewal projects or new construction, which require multi-year planning and efforts.

Phase 3—Long-term (11–15 years): Price subsidies and quality upgrades are effective measures at this phase. These measures primarily aim at ‘matching disadvantage’ CUCs in central urban areas with great demand. It is a long-term effort, since raising elders’ income, lowering institutions’ prices, and improving their quality cannot happen in a short time frame.

The three phases are ordered by dependency and relative cost. The minimum service level demands minimal resources and a low cost; the bed expansion yields the largest equity improvement (Gini reduction of 0.050, the largest among all scenarios) but requires substantial investment; and the subsidies and quality upgrades are of medium cost and will be more effective once the supply is sufficient. This is why we placed subsidies and quality upgrades in the last phase, as they are built upon the second phase (bed expansion).

### 5.3. Scientific Merits of Our ‘4A’ Model

While the optimization models and inequity metrics are individually standard, the contribution of our research does not lie in creating new optimization or inequity metrics. Instead, its significance lies in integrating matching across multiple dimensions, operational constraints, and policy simulation into a single coherent allocation framework. Such a framework outputs concrete bed allocation plans, not just accessibility indices or facility locations. Under the current supply-tight scenario, our ‘4A’ model successfully identifies major issues facing Guangzhou: the ‘scale disadvantage’ + ‘matching disadvantage’ in central urban districts and ‘supply vacuum’ in peripheral suburban areas. This finding indicates that total bed capacity and spatial mismatch play a primary role in the optimization, while affordability and quality are secondary, which does not diminish the importance of affordability and quality. Rather, it highlights our model’s capability to uncover real problems. Our ‘4A’ model provides a quantitative tool for diagnosing the three mechanisms of CUCs that traditional spatial accessibility analysis cannot capture. Furthermore, under a higher matching standard ($M_{ij}\geq 0.3$), our ‘4A’ model sustains a satisfaction rate of 68.7%, but the ‘distance-only variant’ model drops to 63.3%, because it retains many O-D pairs with moderate spatial accessibility but good affordability or quality. The ‘4A’ model can facilitate allocations for communities/villages with low spatial accessibility, and good affordability and quality, avoiding the selection of expensive and low-quality options.

Although we applied the ‘4A’ model to Guangzhou, it is transferable to smaller cities, rural regions, or other countries. The generalizability of Guangzhou-based findings to other cities should be carefully examined. In smaller cities or rural areas, a lack of detailed input data (e.g., community-level income and nursing home prices) may reduce the reliability of findings. Moreover, key parameters (e.g., distance thresholds, weights, and demand ratios) should be adjusted to reflect local healthcare systems, demographic characteristics, or policy environments. For instance, smaller cities or rural regions may have different healthcare systems; their informal family care facilities may be more dominant than formal ones; if only formal facilities are considered, the optimization accuracy may be compromised. Thus, the ‘4A’ model is adaptable to other urban and rural settings, but key parameters are place-dependent and require local adjustments.

The novelty summarized in Section 2.5 (Table 1) has both theoretical and computational implications. Theoretically, studies using 2SFCA (and its variants) and gravity models focus almost exclusively on spatial distance, whereas our ‘4A’ model integrates affordability and acceptability as core decision factors beyond spatial distance; this integration is important as it acknowledges that access to elderly care is shaped by more than just spatial proximity. Computationally, our model serves a different purpose than location-allocation models. It does not seek optimal sites for new facilities. Instead, it allocates beds within existing nursing homes under capacity, demand, and occupancy constraints. The output is a concrete allocation matrix, not accessibility indices, choice probabilities, or facility locations; this distinction makes our model methodologically novel.

### 5.4. Limitations and Future Directions

Despite the value of our ‘4A’ model, several limitations should be acknowledged. These limitations can be grouped into three categories, as described below: First, we have data-related limitations. We excluded the accommodation dimension (e.g., operating hours, room types, and disability support) due to data unavailability. The missing star ratings for 191 nursing homes were imputed based on ownership type; a sensitivity analysis (Appendix A, Table A4) shows that moderate adjustments (±20%) to the imputed scores do not alter the core conclusions. We used community/village average income as a proxy for paying ability in the affordability measure, but ignored pensions, insurance, and subsidies due to a lack of relevant data. Nursing home price data were obtained from a third-party provider, and their data accuracy was not independently verified. Second, we have modeling-related limitations. The matching function assumes linearly additive relationships among all dimensions; nonlinear interactions (e.g., quality compensating for distance) are not captured. A static allocation model was adopted without considering the bed turnover or queuing. We employed the network distance rather than travel time. The effective capacity range was set based on practical reasoning rather than formal empirical validation. In addition, the optimization results were not validated against actual nursing home admission records or historical occupancy patterns, as such data were not available; formal validation is needed in future work. Third, we have scenario-specific limitations. The target bed expansion scenario assumes existing facilities can expand without considering land or staffing constraints.

We acknowledge that our single-objective design does not capture the full trade-off between the four dimensions. Future studies could explore multi-objective methods (e.g., epsilon-constraint) to address this limitation. We should also incorporate individual-level data (e.g., disability status, social networks, pensions, and insurance), nursing home accommodation data (e.g., operating hours, room types, and disability support), dynamic demand forecasting, alternative socioeconomic variables, mode-specific travel times, and nonlinear or machine-learning approaches in future studies. A formal cost-effectiveness analysis using real cost data and dynamic simulation would enhance the policy evaluation. The weights in the matching function were chosen through sensitivity analysis; alternative weighting methods (e.g., expert elicitation and analytic hierarchy process) could refine the selection of weights. The effective capacity range would be formally verified using actual facility-level occupancy data. While we performed separate sensitivity analyses (e.g., matching thresholds, weights, demand ratios, and quality scores) in the current study, a multi-parameter uncertainty quantification (e.g., Monte Carlo simulation) would strengthen the findings in future work. Other factors, including patient health status, family preferences, cultural considerations, and transportation availability, could be explored when the data become available. We also see the potential to use advanced AI and machine-learning techniques (e.g., demand forecasting, and reinforcement learning) for supporting dynamic resource allocation under changing demographic conditions.

## 6. Conclusions

Three main contributions emerge from this research. First, unlike most location-allocation models that focus on locating new facilities, our ‘4A’ model allocates beds from existing nursing homes under demand, capacity, and occupancy constraints. Second, we integrate non-spatial dimensions (affordability and quality) into the objective function besides the spatial measures. Third, we incorporate operational constraints, including minimum and maximum occupancy rates, ensuring that allocation plans are practically implementable. Note that our model is a single-objective optimization framework, as clarified in Section 2.2.

We took Guangzhou as a case study to construct and validate a ‘4A’ allocation model. There are three key findings: (1) 753 communities/villages (28.6%) do not receive any beds, forming a ‘dual-center’ deprivation pattern: a high unmet demand exists both in central urban districts and peripheral suburbs. Further discussion reveals that the formation mechanism in central urban areas is driven by a ‘scale disadvantage’ and ‘matching disadvantage’, whereas the mechanism in peripheral areas is attributable to a ‘supply vacuum’. (2) The overall inequity is moderate (Gini = 0.330), and 92.9% of the inequity originates from within-district disparities. (3) Policy simulations show that a minimum service level (α = 0.1) eliminates all unserved communities/villages without additional beds; targeted bed expansion (+20% within 5 km of CUCs) increases satisfaction from 68.7% to 74.8% and reduces the Gini from 0.330 to 0.280; and price subsidies and quality upgrades show limited effects under resource scarcity but become effective when the bed supply increases by ≥30%.

These findings illustrate the merits of the ‘4A’ model, and its significance is summarized in three aspects: First, it combines affordability and quality with spatial access and operational constraints within a single framework, rather than focusing solely on geographic distance. Second, it generates actual bed allocation plans instead of accessibility scores or new facility locations; the outputs are directly useful for planning. Third, when applied to Guangzhou, the model identifies that unmet demand is driven by different mechanisms: scale and matching disadvantages in central districts, and a supply vacuum in peripheral suburbs. These mechanisms help diagnose why some communities/villages have zero allocations, which a purely spatial analysis cannot capture. The framework can be adapted to other cities.

Like any other empirical study, our model has several limitations, including data-, model-, and scenario-related issues. Future research could incorporate individual-level data, dynamic demand forecasting, and multi-objective techniques to further explore trade-offs among the ‘4A’ dimensions. These limitations and directions are discussed in detail in Section 5.4.

## Figures and Tables

**Table 1:** Comparison of the ‘4A’ model with three existing method types.

Method	Output	Price and Quality	Constraints	Allocation Plan
2SFCA and variants	Accessibility indices	No	No	No
Gravity models	Choice probabilities	No	No	No
Location-allocation models	Facility locations	Rarely	Capacity	No
‘4A’ model	Allocation matrix	Yes	Yes (capacity, demand, occupancy bounds)	Yes

**Table 2:** Baseline model results.

AB	ED	SR	#CUCs	#NCCs
48,553	70,686	68.70%	753 (28.60%)	15

Note: AB, allocated beds; ED, effective demand; SR: satisfaction rate; #, count; CUCs, completely unserved communities/villages; NCCs, no-connection communities/villages.

**Table 3:** Comparison between the ‘4A’ and ‘distance-only variant’ models.

	AB (‘4A’ vs. ‘DV’)	SR (‘4A’ vs. ‘DV’)	# CUCs (‘4A’ vs. ‘DV’)	Gini (‘4A’ vs. ‘DV’)
0.0	48,553 vs. 48,553	68.7% vs. 68.7%	753 vs. 721	0.330 vs. 0.384
0.3	48,553 vs. 47,137	68.7% vs. 66.7%	753 vs. 772	0.330 vs. 0.387
0.5	48,553 vs. 44,740	68.7% vs. 63.3%	753 vs. 869	0.330 vs. 0.403

Note: Gini: Gini coefficient; DV: ‘distance-only’ variant. Other abbreviations (AB, SR, and CUCs) and symbol (#) are as defined in Table 2.

**Table 4:** Results of the four policy simulations under the resource-scarce condition.

Scenario	AB	SR	Gini	#CUCs	ΔAB	ΔSR	ΔGini	Δ#CUCs
Baseline	48,553	68.70	0.330	753	—	—	—	—
Minimum service level (α = 0.1)	48,553	68.70	0.316	0	0	0.00	−0.014	−753
Target bed expansion (+20%)	52,891	74.80	0.280	632	4338	6.11	−0.050	−121
Price subsidy	48,553	68.70	0.318	721	0	0.00	−0.013	−32
Quality upgrade	48,905	69.20	0.323	738	352	0.50	−0.008	−15

Note: Δ, change; #, count; Δ#, change in the count. “—” denotes not applicable. Other abbreviations are as defined in Table 2 (AB, SR, and CUCs) and Table 3 (Gini).

**Table 5:** Different types of CUC and their mechanisms.

Type	Locations	Mechanism	Characteristics
Supply vacuum	Peripheral areas	No nearby facilities	Long distance, facility scarcity
Scale disadvantage	Outer fringe of central urban districts	Small effective demand	Small elderly population; out-competed despite nearby facilities
Matching disadvantage	Inner core of central urban districts	Large effective demand	Large elderly population; low income; high prices, poor quality

## Data Availability

The original and generated data are available upon request.

## Appendix A

Table A1 reports the sensitivity analysis of six weight configurations. It confirms that our baseline weights (0.2, 0.4, and 0.4) achieve the optimal balance between satisfaction (68.70%) and inequity (Gini = 0.330). Entropy weighting [59] was also tested but not adopted.

**Table A1:** Results of weight sensitivity analysis.

Configuration	Weights (Spatial Accessibility, Affordability, Quality)	AB	SR	Gini	#CUCs
Baseline	(0.2, 0.4, 0.4)	48,553	68.70%	0.330	753
Spatial accessibility only	(1.0, 0.0, 0.0)	48,553	68.70%	0.384	721
Affordability only	(0.0, 1.0, 0.0)	45,743	64.70%	0.504	1268
Quality only	(0.0, 0.0, 1.0)	45,743	64.70%	0.319	650
Affordability + Quality balanced	(0.0, 0.5, 0.5)	48,553	68.70%	0.331	756
Spatial accessibility + Affordability + Quality balanced	(0.333, 0.333, 0.333)	45,743	64.70%	0.381	869
Entropy weighting (objective)	(0.944, 0.017, 0.040)	45,743	64.70%	0.416	856

Note: Entropy weighting produced near-spatial weights (0.944, 0.017, and 0.040) and performed worse (Gini 0.416 vs. 0.330; CUCs 856 vs. 753) than our baseline (0.2, 0.4, and 0.4). Therefore, we chose the baseline weight. AB, allocated beds; SR, satisfaction rate; #, count; CUCs, completely unserved communities/villages; Gini: Gini coefficient.

Table A2 summarizes the marginal effects of a price subsidy and quality upgrade under various resource-abundant conditions.

**Table A2:** Results of price subsidy and quality upgrade under resource-abundant conditions.

Resource Level	BM	Scenario	AB	SR	Gini	#CUCs	ΔAB	ΔSR	ΔGini	Δ#CUCs
Moderately abundant	120%	Baseline	58,201	82.34	0.213	441	—	—	—	—
		Price subsidy	58,201	82.34	0.206	423	0	0	−0.007	−18
		Quality upgrade	58,622	82.93	0.205	425	421	0.59	−0.008	−16
More abundant	130%	Baseline	63,042	89.19	0.159	293	—	—	—	—
		Price subsidy	63,042	89.19	0.153	273	0	0	−0.006	−20
		Quality upgrade	63,498	89.83	0.153	283	474	0.64	−0.006	−10
Very abundant	140%	Baseline	67,913	96.08	0.121	195	—	—	—	—
		Price subsidy	67,913	96.08	0.107	154	0	0	−0.014	−41
		Quality upgrade	68,405	96.77	0.110	172	492	0.69	−0.011	−23

Note: BM, bed multiplier; Δ, change; #, count; Δ#, change in the count. “—” denotes not applicable. Other abbreviations are as defined in Table A1.

To evaluate the sensitivity of the 3% demand assumption, we tested alternative demand ratios (2%, 2.5%, 5%, and 10%). Table A3 summarizes the results.

**Table A3:** Results of sensitivity analysis on alternative demand ratios.

DR	ED	AB	SR	Gini	#CUCs
2%	47,124	47,124	100%	0.052	0
2.5%	58,905	48,553	82.40%	0.213	442
3% (baseline)	70,686	48,553	68.70%	0.330	753
5%	117,809	48,553	41.20%	0.600	1474
10%	235,619	48,553	20.60%	0.785	1956

Note: DR, demand ratio; ED (effective demand), total elderly population × DR. Other abbreviations are as defined in Table A1.

The sharp change between 2% and 3% reflects a threshold effect. At 2%, the effective demand is below the effective bed capacity; all demand is satisfied. At 2.5% and above, the demand exceeds the supply, resulting in unmet demand. The main conclusions (the dual-center pattern, three CUC mechanisms, and three-phase plan) remain unchanged across all alternative demand ratios.

To assess the uncertainty arising from the imputed quality scores, we adjusted the imputed values for the 191 nursing homes without star ratings by ±20% and re-ran the optimization. See Table A4. The allocated beds (AB) varied by ±2786 (±5.7%), satisfaction rate (SR) by ±4%, Gini by ±0.04, and CUCs by ±110. The dual-center pattern, the three CUC mechanisms, and the three-phased plan remained robust.

**Table A4:** Sensitivity analysis of imputed quality scores (±20% adjustments).

	AB	SR	OR	Gini	#CUCs
Baseline	48,553	68.70%	0.843	0.330	753
+20% adjustment	51,336	72.60%	0.897	0.298	643
−20% adjustment	45,764	64.70%	0.787	0.374	870
Change (±20%)	±2786	±4.0%	±5.5%	±0.04	±110

Note: OR, occupancy rate. Other abbreviations are as defined in Table A1.

The formulae for spatial clustering and inequality measures in Section 3.5 are provided as follows:

Moran’s I:

(A1) $$I=\frac{n}{\sum_{i=1}^{n}\sum_{j=1}^{n}w_{ij}}\times \frac{\sum_{i=1}^{n}\sum_{j=1}^{n}w_{ij}\left(u_{i}-\bar{u}\right)\left(u_{j}-\bar{u}\right)}{\sum_{i=1}^{n}{\left(u_{i}-\bar{u}\right)}^{2}}$$

Getis–Ord Gi*:

(A2) $$G_{i}^{*}=\frac{\sum_{i=1}^{n}w_{ij}u_{j}-\bar{u}\sum_{i=1}^{n}w_{ij}}{s\sqrt{\frac{n\sum_{i=1}^{n}w_{ij}^{2}-{\left(\sum_{i=1}^{n}w_{ij}\right)}^{2}}{n-1}}}$$

Gini coefficient:

(A3) $$G_{i}=\frac{2\sum_{i=1}^{n}ip_{i}}{n\sum_{i=1}^{n}p_{i}}-\frac{n+1}{n}$$

Theil index:

(A4) $$T=\frac{1}{n}\sum_{i=1}^{n}\frac{r_{i}}{\bar{r}}\ln\frac{r_{i}}{\bar{r}}$$

(A5) $$T=T_{between}+T_{within}$$

(A6) $$T_{between}=\sum_{g=1}^{G}\frac{n_{g}}{n}\frac{\bar{r_{g}}}{\bar{r}}\ln\frac{\bar{r_{g}}}{\bar{r}}$$

(A7) $$T_{within}=\sum_{g=1}^{G}\frac{n_{g}}{n}\frac{\bar{r_{g}}}{\bar{r}}T_{g}$$

where $\bar{u}$ is the mean value of $u_{i}$. $w_{ij}$ represents spatial weights based on the k nearest neighbors (k = 12); s is the standard deviation of all unmet demand rates in the study area; $p_{i}$ denotes a per capita matching score for a community/village i; n is the number of communities/villages; T denotes the Theil index; $r_{i}$ is the satisfaction rate of a community/village i, ${r_{i}=assigned}_{i}/d_{i}$; $\bar{r}$ is the average satisfaction rate of all communities/villages; g is the district index, g = 1, 2,…, G; G is the total number of districts in Guangzhou, and G = 11 in this study; $n_{g}$ is the number of communities/villages in district g; $\bar{r_{g}}$ is the average satisfaction rate in district g; $T_{g}$ is the Theil index in district g; $T_{between}$ denotes the between-district inequity; and $T_{within}$ is the within-district inequity.
